# Monthly drought prediction based on ensemble models

**DOI:** 10.7717/peerj.9853

**Published:** 2020-09-08

**Authors:** Muhammad Haroon Shaukat, Ijaz Hussain, Muhammad Faisal, Ahmad Al-Dousari, Muhammad Ismail, Alaa Mohamd Shoukry, Elsayed Elsherbini Elashkar, Showkat Gani

**Affiliations:** 1 Department of Statistics, Quaid-i-Azam University, Islamabad, Pakistan; 2 Faculty of Health Studies, University of Bradford, Bradford, United Kingdom; 3 Department of Geography, Kuwait University, Kuwait, Kuwait; 4 Department of Statistics, COMSATS University Islamabad, Lahore Campus, Lahore, Pakistan; 5 Arriyadh Community College, King Saud University, Riyadh, Saudi Arabia; 6 KSA, Workers University Egypt, Nasr, Egypt; 7 Administrative Sciences Department, Community College, King Saud University, Riyadh, Saudi Arabia; 8 Applied Statistics Department, Faculty of Commerce, Mansoura University, Mansoura, Egypt; 9 College of Business Administration, King Saud University, Muzahimiyah, Saudi Arabia; 10 Bradford Institute for Health Research, Bradford Teaching Hospitals NHS Foundation Trust, Bradford, United Kingdom

**Keywords:** Standardized precipitation index, Equal ensemble drought prediction, Weighted ensemble drought prediction, Conditional ensemble drought prediction, Copulas

## Abstract

Drought is a natural hazard, which is a result of a prolonged shortage of precipitation, high temperature and change in the weather pattern. Drought harms society, the economy and the natural environment, but it is difficult to identify and characterize. Many areas of Pakistan have suffered severe droughts during the last three decades due to changes in the weather pattern. A drought analysis with the incorporation of climate information has not yet been undertaken in this study region. Here, we propose an ensemble approach for monthly drought prediction and to define and examine wet/dry events. Initially, the drought events were identified by the short term Standardized Precipitation Index (SPI-3). Drought is predicted based on three ensemble models i.e., Equal Ensemble Drought Prediction (EEDP), Weighted Ensemble Drought Prediction (WEDP) and the Conditional Ensemble Drought Prediction (CEDP) model. Besides, two weighting procedures are used for distributing weights in the WEDP model, such as Traditional Weighting (TW) and the Weighted Bootstrap Resampling (WBR) procedure. Four copula families (i.e., Frank, Clayton, Gumbel and Joe) are used to explain the dependency relation between climate indices and precipitation in the CEDP model. Among all four copula families, the Joe copula has been found suitable for most of the times. The CEDP model provides better results in terms of accuracy and uncertainty as compared to other ensemble models for all meteorological stations. The performance of the CEDP model indicates that the climate indices are correlated with a weather pattern of four meteorological stations. Moreover, the percentage occurrence of extreme drought events that have appeared in the Multan, Bahawalpur, Barkhan and Khanpur are 1.44%, 0.57%, 2.59% and 1.71%, respectively, whereas the percentage occurrence of extremely wet events are 2.3%, 1.72%, 0.86% and 2.86%, respectively. The understanding of drought pattern by including climate information can contribute to the knowledge of future agriculture and water resource management.

## Introduction

In the last few decades, global warming has become an undeniable fact which causes climate irregularities i.e., extreme weather events and droughts (Bai et al., 2020; Horton et al., 2016; Mishra & Singh, 2010). Drought is the most complicated hazard among other natural disasters (e.g., flood, earthquake, tropical cyclones) and has a huge impact on society, the economy and natural environments, such as reducing air and water quality, and causing erosion, landscaping (dust), and ecological habitat damage (Bonsai & Wheaton, 2005). It is challenging to determine the characteristic of droughts such as beginning, ending, intensity and duration (Vicente-Serrano, Beguería & López-Moreno, 2010). Sharafati & Pezeshki (2020) proposed a robust approach to assess climate change impact variability on future extreme events over Dehbar catchment in Iran. They used the Lars-WG6 model to generate future climate weather variables such as temperature and rainfall depth using five coupled models with several emission scenarios. They concluded that trends in extreme rainfall depth and river discharge is increasing due to climate and inferred that the future extreme rainfall depth at more than 500-year return periods has more variability.

Several drought indices are used for the detection and characterization of drought, i.e., Standardized Precipitation Index (SPI) (Mckee, Doesken & Kleist, 1993), Standardized Anomaly Index (SAI) (Katz & Glantz, 1986), Standardized Precipitation Evapotranspiration Index (SPEI), (Vicente-Serrano, Beguería & López-Moreno, 2010), Standardized Precipitation Temperature Index (SPTI) (Zulfiqar et al., 2019; Zuliqar et al., 2017) and Reconnaissance Drought Index (RDI) (Tsakiris & Vangelis, 2005). SPI is a popular drought index and is used to identify and monitor drought in the past studies (Achour et al., 2020; Bai et al., 2020; Caloiero & Veltri, 2019; Ellahi et al., 2020). During the estimation phase of SPI, the selection of unsuitable probability distribution may provide biased values of SPI (Zulfiqar et al., 2019; Stagge et al., 2015). Zulfiqar et al. (2019) fitted several appropriate probability distributions in the estimation phase of SPI, SPEI and SPTI. The goodness of fit test was used with 5% level of significance to check the appropriateness of candidate probability distribution and the lowest value of Bayesian Information Criterion (BIC) was used for the selection of suitable probability distribution. Mishra & Desai (2005) used the Autoregressive Integrated Moving Average (ARIMA) and the Seasonal Autoregressive Integrated Moving Average (SARIMA) model on the SPI series (1965–2001) in Kansabati River of India to predict the drought for 2–3 month time scale, and also stated that predicted results are consistent with the observed data. Belayneh, Adamowski & Khalil (2016) used the SPI-3 and SPI-6 of Awash River basin in Ethiopia to forecast drought through Artificial Neural Network (ANN), Support Vector Machine (SVM), and Wavelet Neural Network (WNN) model. The ARIMA model was applied to SPI-12 and SPI-24 to forecast drought and the results of the forecast were compared with machine learning models (Belayneh, Adamowski & Khalil, 2016). Among these several models, the WNN performed better based on R square, MAE and RMSE. Choubin, Malekian & Golshan (2016) utilized the rainfall data from January 1967 to December 2009 of four stations to compute the SPI (1–12) months in Iran. They applied the Adaptive Neuro Fuzzy Inference System (ANFIS), M5P model tree and the Multilayer Perceptron (MLP) model to predict the SPI with the incorporation of climate signal. The accuracy results indicated that the performance of MLP model is better in comparison to other models.

Moradkhani & Meier (2010) used two procedures e.g., Linear Regression (LR) and Ensemble Streamflow Prediction (ESP), for the ensemble prediction. The use of a statistical approach with large scale climate variable is effective for the hydrological prediction (Moradkhani & Meier, 2010; Najafi, Moradkhani & Piechota, 2012). Choubin et al. (2019) applied the Multivariate Adaptive Regression Splines (MARS), M5 Model Tree and the Least Squares Support Vector Machine (LSSVM) to predict the streamflow pattern over the Mediterranean region of Turkey. They concluded that the performance of the LSSVM model is better in comparison to other models that utilized the climate information for streamflow modelling. Also, it is stated that the North Pacific (NP) and the East Central Tropical Pacific Sea Surface Temperature (ECTP-SST) damage the streamflow patterns. Choubin et al. (2014) used the rainfall data from January 1967 to December 2009 of four stations in Iran. They used eight most relevant climate indices in the prediction of drought. They observed that the Atlantic surface temperature had the inverse relationship with SPI and Atlantic Meridional Mode (AMM) had the highest correlation. It was concluded that the forecast performance of the Neuro-Fuzzy (NF) model is better as compared to the Stepwise Regression (SR) model at the two-year lag. AghaKouchak (2015) proposed a Multivariate Standardized Drought Index (MSDI) for the ensemble drought prediction in Africa. In their study, firstly the ESP procedure was applied on the monthly rainfall series and soil moisture series to predict the seasonal changes. The estimation of MSDI is based on the joint probability of accumulated predicted seasonal changes of rainfall and soil moisture from ESP procedure. The ensemble model estimates the amount of severe drought and provide information about their probability of occurrence and results were found to be consistent with the observed data. Bradley, Habib & Schwartz (2015) applied a bootstrap sampling resampling procedure on the daily flow time series (1992–2010) of the Blue Nile river of Ethiopia to produce the ensemble stream flow forecast. The results of the technique were compared with the traditional weighting approach. Moreover, the traditional procedure utilized the Gaussian kernel function for the weighting scheme, whereas the sampling resampling approach uses the concept of Bayesian updating (Smith & Gelfand, 1992). Both techniques assigned weights to each ensemble member in a dissimilar way, but the results of Bayesian updating was found to be better. Zhang, Dong & Chen (2019) proposed three ensemble models, i.e., Equal Ensemble Drought Prediction (EEDP), Weighted Ensemble Drought Prediction (WEDP) and Conditional Ensemble Drought Prediction (CEDP) to forecast the monthly droughts of 26 meteorological stations in Jiangxi province of China. As a result, the CEDP model was found to be better in parameter estimation and accuracy than other models. The choice of climate variables is based on the strong correlation between the forecast variable and climate variable (Najafi, Moradkhani & Piechota, 2012; Zhang, Dong & Chen, 2019; Sharafati, Nabaei & Shahid, 2020) and further climate variables were combined into one Integrated Climate Predictor (ICP) through linear regression model. The hydrological variables do not fulfill the requirement of independence and normality. Zhang & Singh (2006) fitted the joint frequency distribution of flood peak, volume and duration using the data of Amite River Basin, Louisiana, USA. Several techniques were applied to obtain the conditional returned periods such as Archimedean copula, Gumbel mix, and bivariate normal Box–Cox transformation. The copula distribution was found to be better in frequency estimation than others. The choice of suitable copula family is problematic, especially the hydrological variables provide imprecise information about the dependence structure (Renard & Lang, 2007). Therefore, the appropriate bivariate copula was fitted between the climate index and the rainfall series in Jiangxi province of China. The choice of appropriate copula family i.e., Frank, Clayton, Gumbel and AMH copula were based on the minimum value of BIC. Among these copula families, the performance of the Frank copula was better in comparison to others in the copula family (Zhang, Dong & Chen, 2019). Nabaei et al. (2019) have made a comprehensive spatial drought analysis to observe the joint return periods of droughts by using Copula. They applied Archimedean Copulas on various combinations of three characteristics of meteorological droughts (Severity (S), Peak (P) and Duration (D)) and concluded that the Gumbel Copula was the most appropriate for S-P according to the Copula Information Criterion, while the Clayton was most appropriate for S-D and P-D.

Changes in the weather pattern have become a serious problem for the biodiversity, hydrology, water resources, agriculture, forestry, human and livestock health (Farooqi, Khan & Mir, 2005). A drought analysis with the incorporation of climate information has not yet been undertaken in this study region. Therefore, the EEDP, WEDP and CEDP models have been used for describing the effect of climate indices on the drought condition. This study aims to describe and classify the short term wet and dry events. Further, these events will be examined, to find out the most occurring event.

## Materials and Methods

### Study area

**Figure 1:**
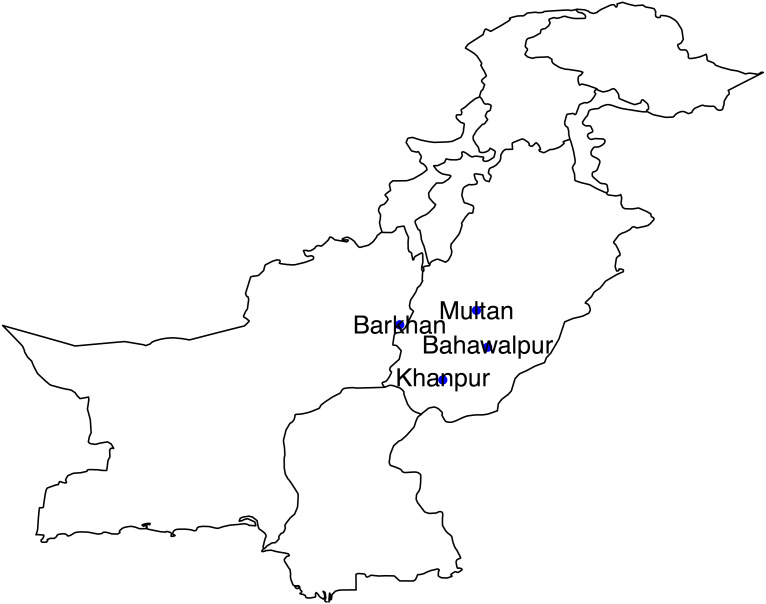
Locations of selected meterological stations.

**Figure 2:**
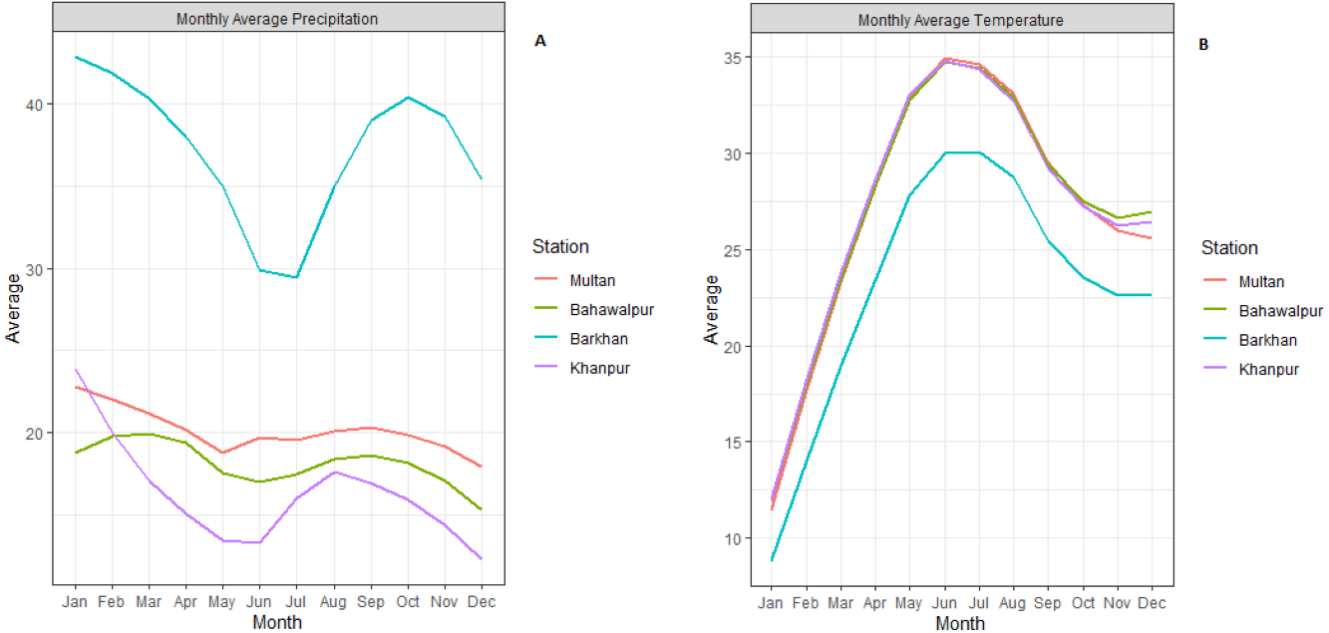
Monthly average precipitation (A) and temperature (B) plots for all meteorological stations.

The study area consists of four meteorological stations of Pakistan (Multan, Bahawalpur, Barkhan and Khanpur). These selected stations are located in the southern part of Pakistan, mostly very hot and mildly cold areas. According to the Meteorological Department of Pakistan, climate change in recent years has led to severe droughts in Southern Pakistan. Agricultural sector plays a leading role in Pakistan’s economy. These stations are popular based on the agriculture sector. So, these stations have been selected based on the agriculture sector and also located in the Southern region (see Fig. 1). For the statistical analysis, the monthly quantitative data of temperature and precipitation from January 1990 to December 2018 has been obtained from the Karachi Data Processing Center through the Pakistan Meteorological Department, Karachi. Initially, the monthly average precipitation and temperature are computed for all stations which are depicted in Fig. 2.

According to (PMD, 2019), many climate indices are the reason for climate change in Southern Pakistan. So, the monthly data of eleven climate indices (Sea Surface Temperature) from January 1990 to December 2018 has been obtained from the Source: https://www.esrl.noaa.gov/psd/data/climateindices/list/. A detailed description of climate indices is described in Table 1.

**Table 1:** Eleven climate indexes (Sea Surface Temperature) are described.

**Climate index**	**Abbreviation**
East pacific/North Pacific Oscillation	EP/NP
Tropical Northern Atlantic Index	TNA
Western Pacific Index	WP
Atlantic Meridional Mode	AMM
Pacific North American Index	PNA
Tropical Northern Atlantic Index	TNA
Artic Oscillation (AAO)	AO (AAO)
Central Tropical Pacific SST (Nino4)	CTP (Nino4)
North Pacific Pattern	NP
Antarctic Oscillation (AAO)	AO/AAO
Southern Oscillation Index	SOI*

### Standardized precipitation index

McKee, Nolan & Kleist (1993) proposed a Standardized Precipitation Index (SPI) for defining and monitoring of wet and dry events i.e., beginning, ending and intensity. The SPI is used to measure the precipitation shortage from the long-term historical record of precipitation and represents the quantitative definition of droughts on multiple time scales i.e., 3, 6, 9, 12, 24 and 48 months. Among these time scales, 3-months SPI is used for the short term, 6-months SPI is used for medium-term and 48-months SPI is used for long term drought analysis (Wu et al., 2007).

In this paper, the cumulative precipitation series such as 3 months’ time scale is used to calculate SPI. This cumulative precipitation time series is utilized to examine the appropriate probability distribution. In the next stage, the appropriateness of candidate probability distributions is checked by Kolmogorov Simonov (KS) (Justel, Peña & Zamar, 1997) and Anderson Darling (AD) ( Anderson & Darling, 1952) test. We used the Easy fit (Schittkowski, 2002) software to compare the goodness of fit measure of several probability distributions. The selection of suitable probability distribution is identified based on the lowest value of BIC. After this, the parameters of each fitted probability distribution are utilized to compute the Cumulative Distribution Function (CDF). In various cases, precipitation time series consist of zero values, due to this reason CDF could be undefined at *y* = 0. For example, we fit the gamma distribution over a precipitation time series and the CDF of gamma distribution could be undefined at *y* = 0. According to Zulfiqar et al. (2019) and Mishra & Desai (2005), an equation is used for this purpose that is given below:

(1) \begin{eqnarray*}G(y)=q+(1-q)F(y)\end{eqnarray*}

where, *q* represents the probability of zero precipitation in time series, while *F*(*y*) is the CDF of a selected probability distribution.

(2) \begin{eqnarray*}q= \frac{m}{n} \end{eqnarray*}

where, *m* is the number of zeros and *n* represents the total number of observations in the precipitation time series. Following Mckee, Doesken & Kleist (1993) and Mishra & Desai (2005), applying the procedure of approximate conversion developed by Abramowitz & Stegun (1948) that converts the CDF into a standard normal variable *Z* with mean zero and unit variance.

The SPI index is as follows:

If,

\begin{eqnarray*}0\lt G(y)\lt 0.5 \end{eqnarray*}

(3) \begin{eqnarray*}t=\sqrt{\ln \nolimits \left[ \frac{1}{{ \left( G(y) \right) }^{2}} \right] }\end{eqnarray*}

Then, it will be used,

(4) \begin{eqnarray*}SPI=Z=-(t- \frac{{c}_{0}+{c}_{1}t+{c}_{2}{t}^{2}}{1+{d}_{1}t+{d}_{2}{t}^{2}+{d}_{3}{t}^{3}} )\end{eqnarray*}

Otherwise,

\begin{eqnarray*}0.5\lt G(y)\lt 1 \end{eqnarray*}

(5) \begin{eqnarray*}& t=\sqrt{\ln \nolimits \left[ \frac{1}{{ \left( 1-G(y) \right) }^{2}} \right] }\end{eqnarray*}

(6) \begin{eqnarray*}& SPI=Z=+(t- \frac{{c}_{0}+{c}_{1}t+{c}_{2}{t}^{2}}{1+{d}_{1}t+{d}_{2}{t}^{2}+{d}_{3}{t}^{3}} )\end{eqnarray*}

where,

c_0_ = 2.515517 c_1_ = 0.802853 c_2_ = 0.010308

d_1_ = 1.432788 d_2_ = 0.189269 d_3_ = 0.001308.

In both cases, these multipliers are used. According to Edwards & McKee (1997) and Mckee, Doesken & Kleist (1993), the classification of SPI is presented in Table 2.

**Table 2:** Drought classification based on standardized precipitation index.

SPI value	Class
>2	Extremely wet
1.5–1.99	Very wet
1.0–1.49	Moderately wet
−0.99 to 0.99	Near normal
−1 to −1.49	Moderately dry
−1.5 to −1.99	Severely dry
< −2	Extremely dry

The positive values of SPI are indicating wet conditions with greater than median precipitation. Whereas, the negative values of SPI are indicating dry conditions with lower than median precipitation. The Standardized Precipitation Index (SPI) is a versatile tool in drought monitoring and analysis. The climatologists around the world had extensively used it for monitoring droughts. SPI is a simple index and only one indicator precipitation is required for the computation. SPI is also used in operational monitoring systems in various countries around the world and is recognized by the World Meteorological Organization (WMO). So, the authors mostly consider SPI based on these features.

### Ensemble models

Zhang, Dong & Chen (2019) proposed ensemble models for the drought prediction that is based on the concept of Ensemble Steamflow Prediction (ESP) model (Liu & Hwang, 2015). The ESP model is a hydrological model that incorporates weather and climate information to represent weather uncertainty in its prediction (Stedinger & Kim, 2010). Ensemble models are divided into three categories based on several weighting techniques that are the EEDP, WEDP and CEDP model.

#### Ensemble streamflow prediction model

ESP model uses the three types of series such as temperature, precipitation and SPI. The aim of using the ESP model is to assess monthly dry conditions. The inclusion of dependence structure (between precipitation and SPI) in the ESP model can strengthen the statistical drought analysis and are useful for estimating inter-annual variability. The choice of the precipitation series is based on the strong relationship between SPI and precipitation. Due to strong correlation, which potentially allows authentic monthly prediction of precipitation that improves monthly SPI prediction. The detailed methodology of initial mean prediction and ensemble mean prediction is described in Liu & Hwang (2015). Moreover, the statistical analysis has been done by the R package ‘*locfit*’ (Loader, 2011). In the ESP model, the prediction of temperature, precipitation and SPI is required. Hydrological time series include various components such as periodically, serial correlation and this feature can be described only by ARIMA and SARIMA models Mishra & Desai (2005).

##### Non-seasonal model (ARIMA).

Geurts (1977) provide a new forecasting tool known as ARIMA model. It is a modification of the ARMA model, which deals with non-stationary series. According to Mishra & Desai (2005), the general ARIMA model is as follows,

(7) \begin{eqnarray*}\varphi (\beta ){\nabla }^{d}{y}_{t}=\theta (\beta ){c}_{t}\end{eqnarray*}

where, *φ*(*β*) is polynomial of Autoregressive (AR) model of order *p*, *θ*(*β*) is polynomial of the moving average model of order *q* and ∇^*d*^ is *d*^*th*^ differencing operator.

##### Seasonal model (SARIMA).

The SARIMA model is a generalization of the ARIMA model. The major advantage of using the SARIMA model is that it deals with the non-stationary series as well as the seasonal series. According to Durdu (2010) and Mishra & Desai (2005), the general SARIMA model is represented as,

(8) \begin{eqnarray*}{\varphi }_{p}(\beta ){\phi }_{p}({\beta }^{s}){\nabla }^{d}{\mathop{\nabla \nolimits }\nolimits }_{s}^{D}{y}_{t}={\theta }_{q}(\beta ){\Theta }_{Q}({\beta }^{s}){c}_{t}\end{eqnarray*}

where *p* and *q* are the order of non-seasonal part of AR and MA and *d*represents the non-seasonal differencing parameter. Besides, *P* and *Q* are the order of seasonal part of the AR and MA model, *D* is a seasonal differencing parameter and *s*represents the length of the season.

The development of time series model includes three stages e.g., identification, estimation of parameters and diagnostic check. Initially, the normality assumption of the time series is checked. In case of non-normal series, Box–Cox transformation and log transformation are applied to satisfy the normality condition. After this, Autocorrelation Function (ACF) and Partial Autocorrelation Function (PACF) were used to scrutinize the structure of transformed series and this information is helpful for fitting the appropriate model. So, the initial parameters have been estimated visually from the plot of ACF and PACF of the time series. Then, the parameters of the model have been done by using Maximum Likelihood Estimation (MLE). Various combinations of parameters were used for the fitting of an appropriate model. Among these appropriate models, the choice of a suitable model was based on the minimum value of AIC. Further, the normality condition of residuals was checked by histogram and normal probability plot. We use the R package ‘forecast’ for the time series modelling.

#### Equal ensemble drought prediction model

The concept of EEDP model is based on the ESP model. In the EEDP model, the historical series that is assumed to have equal weights in the future. The climate series contains the *n* years for a study area so that the number of ensemble members are corresponding to the historical years in the time series and can be generated.

Therefore, the ensemble members of the target month are as follows:

(9) \begin{eqnarray*}SP{I}_{n+1}=(SP{I}_{n+1}^{1},SP{I}_{n+1}^{2},\ldots ,SP{I}_{n+1}^{n}).\end{eqnarray*}

EEDP model assigns equal weights (1∕*n*) to each ensemble member of the target month because there is no information about the climate change to assign divergent weights (Belayneh, Adamowski & Khalil, 2016).

#### Weighted ensemble drought prediction model

One way to represent the impact of climate change is to weight the climate index (Bradley, Habib & Schwartz, 2015). The WEDP model deals with climate index to assign weights to all ensemble members. The aim of assigning weights to each ensemble member is to reflect the change of season when predicted (Belayneh, Adamowski & Khalil, 2016). There are two types of procedure for assigning weights such as Traditional Weighting (TW) and Weighted Bootstrap Resampling (WBR) procedure.

##### Weighted ensemble drought prediction model algorithm.

The procedure for generating ensemble members in WEDP model is similar to that of the EEDP model Eq. (9). According to Zhang, Dong & Chen (2019), WEDP model algorithm is divided into the following steps:

• The appropriate climate index should be chosen based on the strong correlation with the target month of precipitation (*P*_*i*,*j*,*k*_).
• Assign weights to climate indices through linear a regression model to simplify them into one integrated climate predictor (ICP) *φ*.
• Calculate the weight of each ensemble member based on the similarity of climate indices between ICP of observed and predicted year.

##### Traditional weighting procedure.

The impact of climate indices can be signified by an unequal weighting of the ensemble members. Traditional weighting procedure utilizes the Gaussian kernel *K*(*y*) to assign a weight to each ensemble member which is based on the similarity of climate indices between the ICP of predicted and observed year (Belayneh, Adamowski & Khalil, 2016; Najafi, Moradkhani & Piechota, 2012; Zhang, Dong & Chen, 2019). A Gaussian kernel function is used to calculate the climate distance between the ICP of predicted and observed year. Therefore, the weight *w*_*i*_ for *i*th ensemble member is as follows:

(10) \begin{eqnarray*}{w}_{i}= \frac{K( \left\vert {\varphi }_{i}-{\varphi }_{n+1} \right\vert )}{\sum _{i=1}^{n}K( \left\vert {\varphi }_{i}-{\varphi }_{n+1} \right\vert )} \end{eqnarray*}

where, *φ*_*i*_ is the ICP of observed year, whereas *φ*_*n*+1_ is ICP of predicted year, which is obtained from a linear regression model. The Gaussian kernel function is defined as:

(11) \begin{eqnarray*}K(y)= \frac{1}{\sqrt{2\pi h}} {e}^{- \frac{{y}^{2}}{2{h}^{2}} }.\end{eqnarray*}

The ‘ *h*’ is the bandwidth kernel parameter and *h* = *σ*_ε_ (*σ*_ε_ represents the variance of error between the ICP of observed year and ICP of the predicted year).

##### Weighted bootstrap resampling procedure.

Smith & Gelfand (1992) proposed the simplest resampling approach known as Weighted Bootstrap Resampling (WBR) procedure. The procedure of WBR is based on random variates via acceptance and rejection method. The procedure of WBR is as follows:

Suppose *φ*_*i*_ *i* = 1, 2, 3, .., *n* are samples from *g* and *f*.

(12) \begin{eqnarray*}{q}_{i}= \frac{f({\varphi }_{i})}{g({\varphi }_{i})} \end{eqnarray*}

and then,

(13) \begin{eqnarray*}{\omega }_{i}= \frac{{q}_{i}}{\sum _{i=1}^{n}{q}_{i}} \end{eqnarray*}

where, *q*_*i*_ is the *i*th ratio between *f* and *g*. Therefore, *w*_*i*_, *i* = 1, 2, …, *n* are the *n* weights which are corresponding to ‘ *n*’ ensemble members and by definition ${\mathop{\sum }\nolimits }_{i=1}^{n}{\omega }_{i}=1$.

#### Conditional ensemble drought prediction model

##### Algorithm of conditional ensemble drought prediction model.

According to Zhang, Dong & Chen (2019), the first and second steps of the CEDP model are similar to WEDP model in section 2.3.3.1. The three to five steps of CEDP model are defined as:

• Use the copula function to fit the bivariate distribution between the ICP (*φ*) and target month of precipitation (*P*_*i*,*j*,*k*_), i.e., *C*(*F*_*φ*_(*φ*), *F*_*P*_*i*,*j*,*k*__(*P*_*i*,*j*,*k*_)).
• Generate conditional precipitation events 1,000 times given the ICP (*φ*) of the observed year through the bivariate copula distribution.
• Use the conditional precipitation events i.e., ${P}_{i,j,k}^{1},{P}_{i,j,k}^{2},\ldots ,{P}_{i,j,k}^{1000}$ to generate the ensemble members of the target month.

##### Copula ensemble.

The basic aim of using the appropriate copula family is to define the dependency structure between ICP and *P*_*i*,*j*,*k*_. The bivariate copula distribution between ICP (*φ*) and target month of precipitation (*P*_*i*,*j*,*k*_) is as follows:

(14) \begin{eqnarray*}C({F}_{\varphi }(\varphi ),{F}_{{P}_{i,j,k}}({P}_{i,j,k}))\end{eqnarray*}

These, *F*_*φ*_(*φ*) and *F*_*P*_*i*,*j*,*k*__(*P*_*i*,*j*,*k*_) are the marginal distribution of copula function. Marginal distribution has ranged between 0 and 1 because, *F*_*φ*_(*φ*) and *F*_*P*_*i*,*j*,*k*__(*P*_*i*,*j*,*k*_) are the CDF of ICP and *P*_*i*,*j*,*k*_ respectively.

According to Zhang, Dong & Chen (2019), the conditional density function is as follows:

(15) \begin{eqnarray*}f({P}_{i,j,k} \left\vert \varphi \right. )= \frac{f(\varphi ,{P}_{i,j,k})}{f(\varphi )} \end{eqnarray*}

where, *f*(*φ*, *P*_*i*,*j*,*k*_) is the joint density distribution between ICP and *P*_*i*,*j*,*k*_. The density *f*(*φ*) is the marginal density distribution of ICP. Therefore, the 1,000 times conditional precipitation events are obtained from conditional density Eq. (15), i.e.,

(16) \begin{eqnarray*}{P}_{i,j,k}^{1},{P}_{i,j,k}^{2},\ldots ,{P}_{i,j,k}^{1000}.\end{eqnarray*}

Finally, the conditional ensemble members are generated by the SPI approach described in section 2.2 on the conditional precipitation events Eq. (16), i.e.,

(17) \begin{eqnarray*}SP{I}_{n+1}=(SP{I}_{n+1}^{1},SP{I}_{n+1}^{2},\ldots ,SP{I}_{n+1}^{1000})\end{eqnarray*}

Copula

In 1959, the general notation of copula was first introduced (Ward, Glorig & Sklar, 1959). A major feature of the Copula function is to examine the dependence structure between the random variables. Following Kao & Govindaraju (2007) and Zhang & Singh (2006), the one-parameter Archimedean copula is expressed as,

(18) \begin{eqnarray*}{C}_{\phi }(p,q)={\psi }^{-1} \left\{ \psi (p)+\psi (q) \right\} 0\lt p,q\lt 1\end{eqnarray*}

The notation *ψ*^−1^(⋅) represents the copula generator and it is a convex decreasing function. The notation *ϕ* is the parameter of Copula function and *ϕ*(1) = 0. In the present study, four families of Archimedean copulas such as Clayton, Joe, Frank and Gumbel are used.

#### Performance measurement tools

##### Accuracy measure tools.

Various tools are used to evaluate the accuracy of predictions (Chen et al., 2014; Pai & Lin, 2005). There are several accuracy measurements tools such as Mean Absolute Error (MAE), Mean Square Error (MSE) and Root Mean Square Error (RMSE), which are scale-dependent measurement tools. Mean Absolute Percentage Error (MAPE) is widely used for measuring the accuracy of predictions of time series models, it is independent of scale. MAPE measures the magnitude of error in term of percentage. According to Parmar & Bhardwaj (2014) and Swanson, Tayman & Bryan (2011), the accuracy measurement tools are described as follows. i.e.,

(19) \begin{eqnarray*}& MAE= \frac{\sum _{i=1}^{n} \left\vert {y}_{obs,i}-{y}_{pred,i} \right\vert }{n} \end{eqnarray*}

(20) \begin{eqnarray*}& MSE= \frac{\sum _{i=1}^{n}({y}_{obs,i}-{y}_{pred,i})^{2}}{n} \end{eqnarray*}

(21) \begin{eqnarray*}& RMSE=\sqrt{ \frac{\sum _{i=1}^{n}({y}_{obs,i}-{y}_{pred,i})^{2}}{n} }\end{eqnarray*}

(22) \begin{eqnarray*}& MAPE= \frac{1}{n} \sum _{1=1}^{n} \left\vert \frac{{y}_{obs,i}-{y}_{pred,i}}{{y}_{obs,i}} \right\vert \end{eqnarray*}

where, *y*_*obs*,*i*_ is the observed value, *y*_*pred*,*i*_ is the predicted value and ***n*** is the length of time series.

Normalize Root Mean Square Error (NRMSE) is a non-dimensional form of RMSE and it is the standardized disaggrement between observed and predicted value. It is used to test the predictive accuracy between observed and predicted value. According to Zhang, Dong & Chen (2019), NRMSE is defined:

(23) \begin{eqnarray*}NRMSE= \frac{RMSE}{{y}_{obs,\max \nolimits }-{y}_{obs,\min \nolimits }} \end{eqnarray*}

where, *y*_*obs*,max_ and *y*_*obs*,min_ are the maximum and minimum value in the observed time series. The smallest value of MAE, MSE, RMSE, MAPE and NRMSE is an indication of better prediction.

##### Uncertainty measure tool (average bandwidth).

In probabilistic prediction, Chen et al. (2014) presented Average Bandwidth (AB) to measure the prediction uncertainty. The AB provides very important information about the prediction uncertainty., AB is defined as follows:

(24) \begin{eqnarray*}AB= \frac{\sum _{i=1}^{n}({Q}_{i}^{{}^{1-a/2}}-{Q}_{i}^{a/2})}{n} \end{eqnarray*}

where, ${Q}_{i}^{{}^{1-a/2}}$ and ${Q}_{i}^{a/2}$ is an indication of quantiles result of *i*th the year and *a* is the level of significance. Therefore, the smallest value of AB is an indication of better prediction.

## Results

Our first objective is to define and monitor the short-term wet and dry events, which is done by SPI-3. In the estimation phase of SPI-3, several distributions are fitted on 3 months aggregated precipitation series (P-3). A detailed description of fitting suitable probability distribution is illustrated in Table 3.

**Table 3:** Several probability distributions are fitted on the P-3 series of four meteorological stations. The CV represents the critical value of KS and AD test.

**Station**	**Distribution**	**Method**	**Parameter**	**BIC**	**KS test/CV**	**AD test/CV**
Multan	Exponential	MOM	*a* = 0.0164	3542.00	0.0313/0.0730	0.4363/2.5018
Bahawalpur	Burr (4P)	MLE	*c* = 994.69, *a* = 1.0053, *b* = 52811.0, *r* = 0.60942	3488.32	0.0324/0.0730	0.5087/2.5018
Barkhan	Fitgue Life (3P)	MLE	*a* = 0.91399, *b* = 82.187, *r* = − 4.0011	3936.46	0.0308/0.0730	0.4367/2.5018
Khanpur	Log normal	MOM	*r* = 3.3099, *b* = 1.0996	3350.00	0.0357/0.0730	0.5202/2.5018

**Notes.**

The *b*, *r* and *k* are the scale, location and rate parameter respectively. As well as both *a* and *c* are the shape parameter.

After this, the cumulative distribution function (CDF) is computed. Later, the CDF of P-3 is utilized for the construction of SPI-3 by following the procedure described in section 2.2.

According to Table 2, all meteorological stations are distressed from various categories of wet and dry events e.g., normal, moderate, severe and extreme. But the occurrence percentage of extreme drought events have appeared in the Multan, Bahawalpur, Barkhan and Khanpur is 1.44%, 0.57%, 2.59% and 1.71% respectively. Whereas, the occurrence percentage of extremely wet events are 2.3%, 1.72%, 0.86% and 2.86% for Multan, Bahawalpur, Barkhan and Khanpur, respectively.

In the ESP procedure, the prediction of Temperature (T), Precipitation (P), P-3 and SPI-3 are needed. In this regard, the T, P, P-3 and SPI-3 are divided into two sets known as a training and testing set. The entire series of T, P, P-3 and SPI-3 are from the year 1990 to 2018. The length of the series in a training set is from the year 1990 to 2011. Whereas, the length of the series in the testing set is from the year 2012 to 2018. The prediction accuracy is measured by several tools i.e., MSE, RMSE, MAE and MAPE. Hence, the detailed summary of time series model fitting is described in Table 4.

**Table 4:** Two-time series models are applied to the P, P-3, T and SPI-3 of all meteorological stations. The term non-seasonal represents the parameters of the ARIMA model while the term seasonal represents the parameters of the SARIMA model.

**Station**	**Series**	**Model**	**Non-Seasonal**	**Seasonal**	**AIC**	**MSE**	**RMSE**	**MAE**	**MAPE**
Multan	T	ARIMA	(1, 0, 0)	(0, 0, 0)	1486.84	0.0114	0.1069	0.0867	0.3781
	SPI-3	ARIMA	(0, 2, 1)	(0, 0, 0)	718.79	0.0000	0.0028	0.0021	0.3294
	P-3	ARIMA	(0, 1, 1)	(0, 0, 0)	883.19	0.0013	0.0355	0.0244	0.9477
	P	ARIMA	(0, 1, 0)	(0, 0, 0)	3585.84	0.0000	0.0000	0.0000	0.0000
Bahawalpur	T	ARIMA	(1, 0, 0)	(0, 0, 0)	1483.58	0.0138	0.1174	0.0947	0.3851
	SPI-3	ARIMA	(0, 2, 1)	(0, 0, 0)	642.33	0.0000	0.0025	0.0020	0.3294
	P-3	SARIMA	(0, 0, 0)	(0, 2, 1)	787.75	0.0002	0.0123	0.0119	0.3294
	P	ARIMA	(0, 1, 0)	(0, 0, 0)	3437.33	0.0000	0.0000	0.0000	0.0000
Barkhan	T	ARIMA	(1, 0, 0)	(0, 0, 0)	1992.95	0.1709	0.4133	0.2339	1.1762
	SPI-3	ARIMA	(0, 2, 1)	(0, 0, 0)	607.19	0.0000	0.0030	0.0023	0.3294
	P-3	SARIMA	(0, 0, 0)	(0, 2, 1)	593.70	0.0002	0.0147	0.0144	0.3294
	P	ARIMA	(0, 2, 1)	(0, 0, 0)	3671.28	0.0254	0.1594	0.0881	0.3187
Khanpur	T	ARIMA	(1, 0, 0)	(0, 0, 0)	1476.00	0.0116	0.1079	0.0848	0.3579
	SPI-3	ARIMA	(0, 2, 1)	(0, 0, 0)	715.24	0.0000	0.0029	0.0024	0.3294
	P-3	SARIMA	(0, 0, 0)	(0, 2, 1)	787.10	0.0001	0.0113	0.0108	0.3294
	P	ARIMA	(0, 2, 1)	(0, 0, 0)	3597.36	0.0000	0.0000	0.0000	0.0000

The selection of P-3 is based on the correlation between P-3 and SPI-3 for all stations. Therefore, the correlation between P-3 and SPI-3 has been found strong in all stations i.e., Multan (0.90), Bahawalpur (0.93), Barkhan (0.93) and Khanpur (0.77). Due to this strong correlation, which permits the reliable monthly predictions of P-3 potentially, which improves the monthly SPI-3 prediction. The selection of the target and the current month has been based on average low precipitation and high temperature of all meteorological stations (Fig. 2). Thus, resulting from the time series models, the observed and predicted values of SPI-3, T and P-3 kept as the current month (April). Whereas, the values of SPI-3, T and P-3 of May, June and July kept as target month. After this, the ensemble members of the target month (i.e., May, June and July) are generated by applying the procedure of initial mean prediction described in section 2.3.1. Therefore, seven ensemble members are generated because the test set consists of seven years of observation, i.e., (2012–2018). In the EEDP model, the equal weights i.e., (1/7 = 0.1428) are distributed to all ensemble members. After this, the ensemble means the prediction of EEDP model is obtained by taking the average of ensemble member. The detailed results of EEDP model are presented in Table 5.

**Table 5:** Equal Ensemble Drought Prediction (EEDP) model results.

**Station**	**Month**	**MAE**	**MSE**	**RMSE**	**NRMSE**	**AB**
Multan	May	0.8377	1.8846	1.3728	0.8351	0.1585
	June	1.0247	1.5137	1.2303	0.9325	0.1532
	July	0.8884	1.2422	1.1145	0.7140	0.1976
Bahawalpur	May	0.9816	1.4192	1.1913	0.4759	0.1388
	June	1.1423	2.2539	1.5013	0.6756	0.1076
	July	0.9788	1.7295	1.3151	0.8470	0.1947
Barkhan	May	1.3706	2.3978	1.5485	0.5375	0.2134
	June	1.0361	1.3170	1.1476	0.3793	0.1714
	July	1.0705	1.4418	1.2008	0.3439	0.1869
Khanpur	May	1.3213	2.7753	1.6659	0.8180	0.1100
	June	1.3191	2.1641	1.4711	0.6950	0.2692
	July	0.9841	1.2493	1.1177	0.7597	0.2142

Table 5 summarizes the statistical results of accuracy and uncertainty of EEDP. The MAE, MSE, RMSE and NRMSE indicate the amount of error between observed and predicted SPI-3 for all the target months of meteorological stations. The prediction uncertainty has been tested by AB at the 5% level of significance. The smallest value of AB indicates less uncertainty in the prediction for all target months of meteorological stations.

Eleven climates indices (sea surface temperature) have been used for weighting the ensemble members of target months (i.e., May, June and July) which is described in Table 2. Initially, the correlation is computed between the climate index and *P*_4,5,6_. Then, three highly correlated climate indices are selected for each target month.

According to Bradley, Habib & Schwartz (2015), select only one climate index to represent climate information. Thus, the LR model has been used to simplify the climate indices into one ICP. Our purpose of climate index prediction is to construct the ICP of observed and predicted year. Further, the estimation of weighting parameters in the LR model is based on the least sum of squared of error between the climate index and *P*_4,5,6_. Two weighting procedures i.e., TW and WBR are used in WEDP model to assign the weights to seven ensemble members. The detailed results of WEDP are presented in Table 6.

**Table 6:** Weighted Ensemble Drought Prediction (WEDP) is obtained by the Traditional Weighting (TW) and Weighted Bootstrap Resampling (WBR) procedure.

**Stations**	**Technique**	**Month**	**MAE**	**MSE**	**RMSE**	**NRMSE**	**AB**
Multan	TW	May	0.8581	1.7314	1.3158	0.8004	0.1585
		June	1.0583	1.5545	1.2468	0.9450	0.1532
		July	0.8680	1.2918	1.1366	0.7281	0.1976
	WBR	May	0.7448	1.1928	1.0922	0.6644	0.1985
		June	0.9614	1.2309	1.1094	0.8409	0.1878
		July	0.7606	0.7472	0.8644	0.5538	0.1722
Bahawalpur	TW	May	0.9611	1.4377	1.1990	0.4789	0.1388
		June	1.1560	2.3033	1.5177	0.6830	0.1076
		July	1.0124	1.7004	1.3040	0.8399	0.2125
	WBR	May	0.9389	1.1797	1.0862	0.4339	0.1260
		June	1.0364	1.4484	1.2035	0.5416	0.1037
		July	1.0501	1.6186	1.2722	0.8194	0.2917
Barkhan	TW	May	1.3502	2.3512	1.5334	0.5323	0.2134
		June	1.0565	1.3745	1.1724	0.3874	0.1714
		July	1.0909	1.4710	1.2128	0.3474	0.1869
	WBR	May	1.1808	1.7197	1.3114	0.4552	0.1718
		June	0.9895	1.1720	1.0826	0.3578	0.1413
		July	0.9577	1.2130	1.1014	0.3155	0.1909
Khanpur	TW	May	1.3054	2.8336	1.6833	0.8265	0.0924
		June	1.1878	2.1540	1.4677	0.6934	0.1611
		July	0.8808	1.2154	1.1025	0.7494	0.2049
	WBR	May	1.2864	2.3129	1.5208	0.7467	0.0935
		June	1.1301	1.9354	1.3912	0.6573	0.2157
		July	0.8082	0.9790	0.9894	0.6725	0.2090

Table 6 showed that the numerical values of MAE, MSE, RMSE and NMSE of WBR procedure are lower as compared to TW procedure for all target months. While the AB indicates that the uncertainty in the prediction of SPI-3 by WBR procedure is higher as compared to TW procedure for some target months.

According to Zhang, Dong & Chen (2019), the log-normal distribution is fitted on the marginal distribution of observed ICP and *P*_4,5,6_ in the construction of the CEDP model. The parameters estimation of log-normal distribution is done by MLE. Their specified distribution is tested by KS and AD test at the 5% level of significance which satisfies the specified distribution. Hence, the description of the log-normal fitting is presented in Table 7.

**Table 7:** Log-normal distribution is fitted on the ICP and *P*_4,5,6_. The CV represents the critical value of KS and AD test.

**Station**	**Month**	**Variable**	**Parameter**	**KS test/CV**	**AD test/CV**
Multan	May	P	*r* = − 0.7430, *b* = 3.5715	0.2888/0.4834	0.5896/2.5015
	May	ICP	*r* = 2.7755, *b* = 0.5200	0.2195/0.4834	0.4733/2.5015
	June	P	*r* = − 1.0108, *b* = 2.5039	0.2288/0.4843	0.5277/2.5018
	June	ICP	*r* = 3.5203, *b* = 0.1075	0.1488/0.4834	0.1782/2.5018
	July	P	*r* = − 0.7430, *b* = 3.5715	0.2888/0.4834	0.5896/2.5018
	July	ICP	*r* = 1.7909, *b* = 0.8844	0.2404/0.4834	0.4290/2.5018
Bahawalpur	May	P	*r* = 0.0394, *b* = 2.5990	0.1835/0.4838	0.3180/2.5018
	May	ICP	*r* = 2.9823, *b* = 0.8369	0.2544/0.4838	0.5062/2.5018
	June	P	r=-0.0916, *b* = 2.3209	0.2310/0.4838	0.4438/2.5018
	June	ICP	*r* = 2.7978, *b* = 0.6074	0.2949/0.4834	0.8844/2.5018
	July	P	*r* = 0.0394, *b* = 2.5990	0.1835/0.4838	0.3180/2.5018
	July	ICP	*r* = 3.0684, *b* = 0.4993	0.2299/0.4838	0.3049/2.5018
Barkhan	May	P	*r* = 2.2407, *b* = 1.4177	0.1799/0.4838	0.2628/2.5018
	May	ICP	*r* = 3.4541, *b* = 1.2763	0.3072/0.4838	0.6141/2.5018
	June	P	*r* = 2.0067, *b* = 0.3757	0.2909/0.4838	0.6682/2.5018
	June	ICP	*r* = 4.1071∕*b* = 0.4194	0.1754/0.4838	0.2233/2.5018
	July	P	*r* = 2.2407, *b* = 1.4177	0.1799/0.4838	0.2628/2.5018
	July	ICP	*r* = 3.4602, *b* = 1.2493	0.1860/0.4838	0.2411/2.5018
Khanpur	May	P	*r* = 1.7262, *b* = 1.19029	0.2260/0.4834	0.2552/2.5018
	May	ICP	*r* = 2.4307, *b* = 0.2685	0.2423/0.4834	0.3672/2.5018
	June	P	*r* = 0.6564, *b* = 2.3527	0.2534/0.4834	0.5869/2.5018
	June	ICP	*r* = 2.7886, *b* = 1.1610	0.3961/0.4834	1.0010/2.5018
	July	P	*r* = 1.7262, *b* = 1.1902	0.2260/0.4834	0.2552/2.5018
	July	ICP	*r* = 2.4341, *b* = 0.2567	0.1566/0.4834	0.1700/2.25018

**Notes.**

b indicates scale parameter and r is the location parameter

However, the estimated parameters are used for the computation of CDF of ICP and *P*_4,5,6_. Besides, four copula families are used i.e., Frank, Joe, Gumbel and Clayton copula for fitting the bivariate distribution between the CDF of observed ICP and *P*_4,5,6_. The parameter estimation of bivariate copula distribution is done by MLE. Also, the choice of suitable copula family is based on the lowest value of BIC. Therefore, the detailed numerical description of copula fitting is presented in Table 8.

**Table 8:** Fitting of suitable copula family for each target month.

**Station**	**Month**	**Family**	**BIC**	**Parameter**
Multan	May	Frank	0.92	−3.0232
	June	Joe	−2.89	3.1327
	July	Frank	1.78	−1.3276
Bahawalpur	May	Joe	−5.71	2.2591
	June	Joe	−6.27	4.6327
	July	Joe	−0.45	1.6008
Barkhan	May	Joe	1.83	1.2306
	June	Joe	−2.58	1.6724
	July	Clayton	0.54	0.9117
Khanpur	May	Frank	0.12	−4.0846
	June	Frank	0.91	−3.1953
	July	Gumbel	−3.41	2.3232

After this, 1,000 times conditionally precipitation events of the *P*_4,5,6_ given the observed ICP are generated. These conditional precipitation events are transformed into SPI by fitting the suitable probability distribution. Their proper fitting is tested through the KS and AD test at the 5% level of significance. Among these appropriate candidate probability distributions, the suitable probability distribution is selected based on the lowest value of BIC. The description of suitable fitted probability distribution on conditional precipitation events is described in Table 9.

**Table 9:** Several suitable probability distributions are fitted on the conditional precipitation event for concerned target month. The CV represents the critical value of KS and AD test.

**Station**	**Month**	**Distribution**	**Method**	**Parameter**	**BIC**	**KS test/CV**	**AD test/CV**
Multan	May	Gen. Extreme value	MLE	*a* = 0.61254, *r* = 0.05452, *b* = 0.09311	−12.28	0.2489/0.4834	0.3677/2.5018
	June	Log normal	MLE	*r* = − 0.49346, *b* = 1.9194	25.98	0.1961/0.4834	0.2875/2.5018
	July	Gen. Extreme value	MLE	*a* = 0.93593, *r* = 0.06398, *b* = 0.17494	0.12	0.2540/0.4834	0.6516/2.5018
Bahawalpur	May	Frechet (2P)	LSM	*a* = 0.33393, *b* = 0.00218	−18.29	0.2103/0.4843	0.3708/2.5018
	June	Gamma	MOM	*a* = 0.64578, *b* = 1.4947	16.20	0.1274/0.4834	0.1786/2.5018
	July	Gamma	MOM	*a* = 0.36233, *b* = 1.7781	6.53	0.1429/0.4834	0.1827/2.5018
Barkhan	May	Gamma	MOM	*a* = 0.65139, *b* = 0.08513	−23.60	0.1280/0.4834	0.1366/2.5018
	June	Frechet (3P)	MLE	*a* = 1.0743, *b* = 0.09702, *r* = 0.03801	1.84	0.2684/0.4834	0.5208/2.5018
	July	Weibull	LSM	*a* = 0.49131, *b* = 0.04994	−20.00	0.1911/0.4834	0.3757/2.5018
Khanpur	May	Gen. Pareto	MLE	*a* = 0.03028, r=-0.00273, *b* = 0.09218	−13.05	0.2527/0.4834	0.5247/2.5018
	June	Burr	MLE	*c* = 3704.5, *a* = 0.81555, *b* = 2967.3	−6.44	0.1910/0.4834	0.4012/2.5018
	July	Gen. Extreme value	MLE	*a* = 0.2735, *r* = 0.03681, *b* = 0.05368	−13.05	0.1602/0.4834	0.2219/2.5018

Lately, CDF of the suitable fitted probability distribution is transformed into 1,000-time conditional ensemble drought member. Finally, the CEDP is obtained by taking the average of conditional ensemble drought member. The prediction accuracy is tested by several tools i.e., MSE, RMSE, MAE and NRMSE. Moreover, the prediction uncertainty is checked at the 5% level of significance. The detailed description of the CEDP model is described in Table 10.

**Table 10:** Conditional Ensemble Drought Prediction (CEDP) model results.

**Station**	**Month**	**MAE**	**MSE**	**RMSE**	**NRMSE**	**AB**
Multan	May	0.672	0.789	0.888	0.540	0.004
	June	0.740	0.775	0.880	0.667	0.006
	July	0.362	0.232	0.481	0.308	0.006
Bahawalpur	May	0.774	0.717	0.846	0.338	0.001
	June	0.423	0.505	0.711	0.320	0.007
	July	0.491	0.489	0.700	0.451	0.005
Barkhan	May	0.684	0.773	0.879	0.305	0.005
	June	0.605	0.851	0.922	0.305	0.004
	July	0.681	1.032	1.016	0.291	0.003
Khanpur	May	0.695	0.708	0.842	0.413	0.006
	June	0.641	0.557	0.747	0.353	0.004
	July	0.438	0.290	0.538	0.366	0.007

From Table 10, it can be observed that numerical values of prediction accuracy (i.e., MAE, MSE, RMSE and NRMSE) and uncertainty (i.e., AB) indicate better prediction. Because, the lowest value of MAE, MSE, RMSE, NRMSE and AB is an indication of better prediction. Moreover, it is an indication of climate indices are correlated with the weather pattern of four meteorological stations.

## Discussion

**Figure 3:**
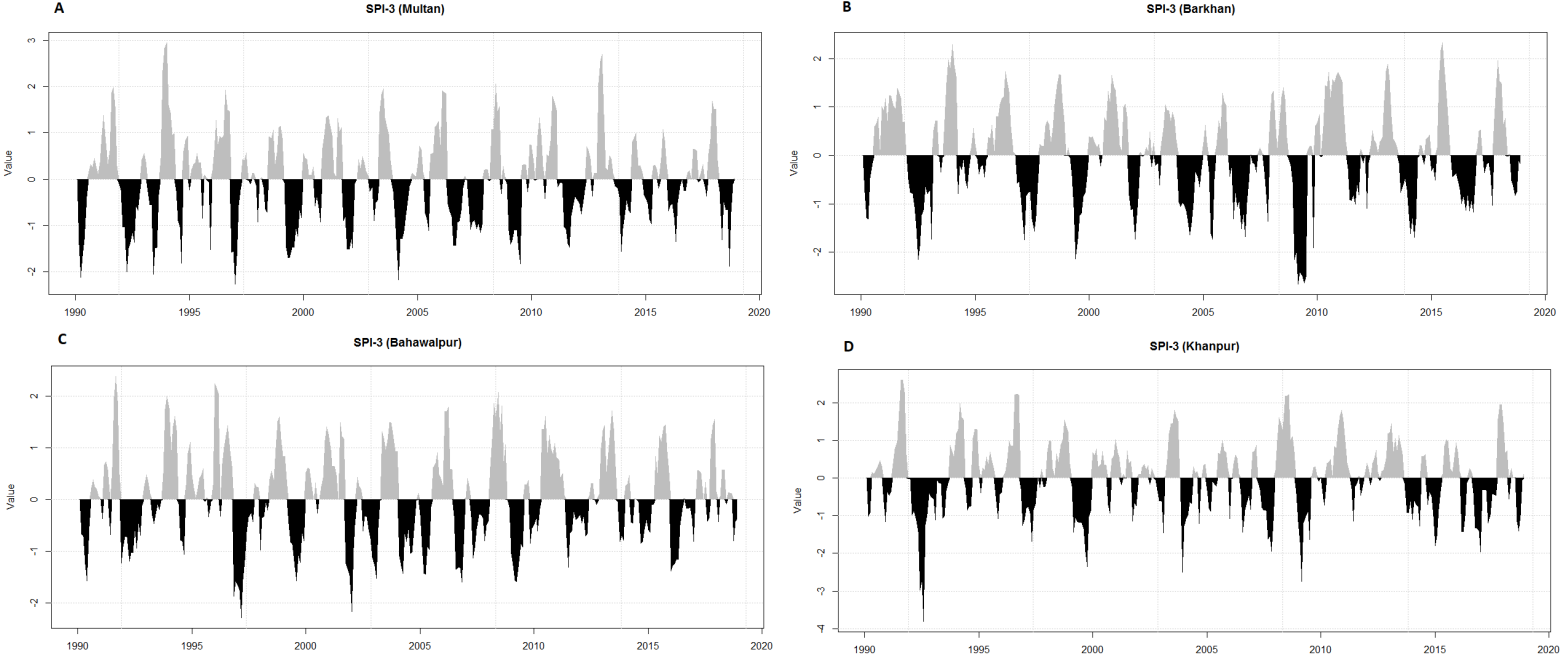
Temporal representation of SPI-3 for four meteorological stations (Multan (A), Barkhan (B), Bahawalpur (C) and Khanpur (D)).

It is challenging to define the features of drought i.e., beginning, ending, intensity and duration (Vicente-Serrano, Beguería & López-Moreno, 2010). Initially, the SPI-3 is used for the definition and monitoring of wet and dry events because the SPI is used in operational monitoring systems in various countries and also acknowledged by the World Meteorological Organization (WMO). It is observed from the temporal plots (Fig. 3), most of the meteorological stations have severely been damaged by various categories of wet and dry events. The highest percentage of extremely dry events is in Barkhan (2.59%) as compared to Khanpur (1.71%), Multan (1.44%) and Bahawalpur (0.57%). In contrast, the high percentage of extremely wet events are found in Khanpur (2.86%) as compared to Multan (2.3%), Bahawalpur (1.72%) and Barkhan (0.86%).

Drought is a complicated phenomenon, so one indicator (i.e., precipitation) may be inadequate to explain the characteristics of drought (AghaKouchak, 2015). The prediction of drought based on the SPI may be insufficient to provide information to overcome drought, so that premature action can be taken. Furthermore, various variables, such as precipitation, climate indices and temperature can be involved in drought conditions in several ways and should also be taken into account in the evaluation and prediction of drought (Hao, Hao & Singh, 2016). In this regard, ensemble models were used which are based on the concept of ESP model that incorporates the weather information in the prediction. In this paper, three ensemble models are used for the drought prediction. Further, two weighting procedures i.e., TW and WBR are discussed in the WEDP model. Among these three models, the EEDP model does not use climate information whereas WEDP and CEDP models incorporate climate information. However, we assigned equal weights to seven ensemble members. The prediction of drought with the incorporation of climate indices is essential and effective.

Eleven climates indices are selected based on correlation with target months of precipitation (Table 11). Then highest correlated climate indices are further simplified into ICP by LR model as the LR model contemplates the importance of climate index to simplifies into climate index. Najafi, Moradkhani & Piechota (2012) were utilized the Spearmen correlation and PCA to consider the predictors. There is one problem that the PCA obtain the information from climate indices to provide in several components. So, it is very hard to fit the joint distribution.

**Table 11:** The CIM represents the selected climate index month, TM represents the target month and the given correlation is representing the relationship between the *P*_4,5,6_ and CIM. These described parameters are of the ARIMA model.

**Station**	**Climate index**	**CIM**	**TM**	**Correlation**	**Parameter**	**AIC**	**MSE**	**RMSE**	**MAE**	**MAPE**
Multan	EP /NP	August	May	0.32	(0, 1, 0)	88.10	7E-33	8E-17	3E-17	3E-15
	TNA	November	May	0.32	(0, 1, 0)	110.03	3E−32	2E−16	9E−17	2E−14
	WP	August	May	0.37	(0, 1, 0)	88.10	7E−33	8E−17	3E−17	3E−15
	AMM	June	June	0.44	(0, 1, 0)	79.02	4E−34	2E−17	8E−18	2E−15
	PNA	May	June	0.4	(0, 1, 0)	96.49	1E−33	3E−17	2E−17	2E−01
	TNA	June	June	0.39	(0, 1, 0)	23.68	2E−34	1E−17	5E−18	1E−14
	AO (AAO)	September	July	0.33	(0, 2, 1)	72.92	4E−33	6E−17	5E−17	1E−14
	CTP (Nino4)	November	July	0.31	(0, 1, 0)	24.39	1E−35	3E−18	1E−18	1E−14
	NP	March	July	0.32	(0, 2, 1)	86.28	1E−33	3E−17	2E−17	2E−14
Bahawalpur	AO (AAO)	September	May	0.35	(0, 1, 0)	72.92	4E−33	6E−17	5E−17	1E−14
	AMM	June	May	0.3	(0, 1, 0)	79.02	4E−34	2E−17	8E−18	2E−15
	EP /NP	August	May	0.32	(0, 1, 0)	88.10	7E−33	8E−17	3E−17	3E−15
	AO/AAO	October	June	0.38	(0, 1, 0)	94.62	2E−33	5E−17	3E−17	1E−14
	AMM	June	June	0.34	(0, 1, 0)	79.02	4E−34	2E−17	8E−18	2E−15
	PNA	May	June	0.42	(0, 1, 0)	96.49	1E−33	3E−17	2E−17	2E−14
	AO/AAO	September	July	0.46	(0, 1, 0)	91.60	5E−34	2E−17	1E−17	4E−15
	NP	February	July	0.35	(0, 1, 0)	110.25	7E−33	9E−17	4E−17	2E−14
	SOI	January	July	0.29	(0, 2, 1)	147.56	6E−03	8E−02	7E−02	4E+00
Barkhan	AO/AAO	September	May	0.48	(0, 1, 0)	103.74	7E−33	8E−17	3E−17	2E−15
	EP /NP	August	May	0.43	(0, 1, 0)	88.10	7E−33	8E−17	3E−17	3E−15
	NP	March	May	0.36	(0, 1, 0)	101.99	4E−33	7E−17	5E−17	2E−14
	AO (AAO)	September	June	0.36	(0, 1, 0)	72.92	4E−33	6E−17	5E−17	1E−14
	EP/NP	May	June	0.35	(0, 2, 1)	105.13	1E−03	4E−02	3E−02	4E+00
	PNA	September	June	0.35	(0, 1, 0)	96.49	1E−33	3E−17	2E−17	2E−14
	CTP (Nino4)	September	July	0.32	(0, 1, 0)	91.60	5E−34	2E−17	1E−17	4E−15
	NP	March	July	0.33	(0, 1, 0)	86.28	1E−33	3E−17	2E−17	2E−14
	PNA	December	July	0.33	(0, 2, 1)	98.35	5E−33	7E−17	5E−17	9E−15
Khanpur	EP /NP	August	May	0.46	(0, 1, 0)	88.10	7E−33	8E−17	3E−17	3E−15
	AO (AAO)	July	May	0.31	(0, 1, 0)	103.47	7E−33	8E−17	3E−17	9E−15
	WP	August	May	0.49	(0, 1, 0)	88.10	7E−33	8E−17	3E−17	3E−15
	AO/AAO	June	June	0.27	(0, 1, 0)	111.90	9E−33	9E−17	5E−17	2E−14
	NP	October	June	0.29	(0, 2, 1)	123.92	2E−03	5E−02	4E−02	4E−03
	PNA	September	June	0.28	(0, 1, 0)	91.60	5E−34	2E−17	1E−17	4E−15
	AO (AAO)	September	July	0.29	(0, 1, 0)	72.92	4E−33	6E−17	5E−17	1E−14
	AMM	February	July	0.3	(0, 1, 0)	110.25	7E−33	9E−17	4E−17	2E−14
	EP /NP	May	July	0.31	(0, 1, 0)	105.13	1E−03	4E−02	3E−02	4E+00

Two weighting procedures, i.e., TW and WBR, used the climate indices in the ensemble prediction. Then the drought is predicted by EEMD, WEDP and CEDP model. It is found that the accuracy and uncertainty which is obtained by TW procedure is like the EEDP model (Tables 5 and 6). Whereas, the results of accuracy which is obtained by WBR is almost better as compared to TW procedure and EEDP model (Tables 5 and 6). But the results of uncertainty indicate similarity to TW procedure and EEMD model. Prediction uncertainty is related to variability. The high variability in prediction may cause of reducing the reliability. The AB is based on the difference between lower and upper quantile. These lower and upper quantiles are associated with the confidence level. According to Xiong et al. (2009), the bandwidth of the prediction bounds is as narrow as possible as it captures the most important information about the prediction uncertainty. In the CEDP model, four Archimedean copula families are used to explain the dependency structure between climate indices and precipitation. The choice of best copula family is based on the lowest value of BIC, and it is observed that Joe copula has been used for most of the times (Table 8). As seen, the results of accuracy and uncertainty indicate CEDP model is better as compared to WEDP and EEDP (Tables 5, 6 and 10). The achieved results of CDEP model indicate that the climate indices are correlated with weather patterns for four meteorological stations.

In TW procedure, we always need the parameter of Gaussian kernel function for computing the weights. The estimation of Gaussian kernel parameter decreases the accuracy of prediction and required more investigation in the computation while the WBR procedure only utilizes the ICP of observed year. The ratios are obtained between ICP of observed year and corresponding ensemble member. The traditional bootstrap resampling procedure is dissimilar from WBR procedure (Burnham & Efron, 1983) because the weight is obtained by dividing the ratio further. The EEDP and WEDP model have generated the ensemble members corresponding to the historical year in series while the generation of ensemble members is not restricted to the historical year in CEDP model. Therefore, the CEDP model is more convenient as compared to the EEDP and WEDP model. But, there is a limitation in the CEDP model. The ensemble prediction by CEDP model required the accurate fit of bivariate copula distribution and probability distribution for conditional precipitation events. In contrast, ensemble prediction will not be accurate. Additionally, these techniques can be used to further improve the study. To deal with stations which are seasonally influenced by droughts, the seasonal drought analysis can be done instead of monthly drought prediction.

## Conclusion

The present analysis consists of four meteorological stations i.e., Multan, Bahawalpur, Barkhan and Khanpur. Initially, SPI-3 was selected to define and classify the drought for all stations. It was observed that the highest percentage of extremely dry events were in Barkhan. In contrast, the high percentage of extremely wet events were found in Khanpur.

Later, three ensemble models are used for the monthly drought prediction. Further, two weighting procedures i.e., TW and WBR were included in the WEDP model. Among these three models, the performance of the CEDP model in terms of accuracy and uncertainty is better in comparison to EEDP and WEDP for all meteorological stations. The performance of the CEDP model indicates that the climate indices are correlated with a weather pattern of four stations as the construction of the CEDP model based on the correlated structure of climate index and precipitation.

##  Supplemental Information

10.7717/peerj.9853/supp-1 Table S1 Geographic informations of selected meterological stations

10.7717/peerj.9853/supp-2 Supplemental Information 2 Data of Bahawalpur Stations

10.7717/peerj.9853/supp-3 Supplemental Information 3 Code

## Additional Information and Declarations

### Data Availability

The following information was supplied regarding data availability: The R code and data from the Bahawalpur stations are available in the Supplemental Files.
