# Supplementary material for: Monthly drought prediction based on ensemble models
Source: PeerJ. 2020 Sep 8;8:e9853. doi: 10.7717/peerj.9853 (PMC7485508; doi:10.7717/peerj.9853)
Supplement: Table S1 [file peerj-08-9853-s001.docx]

Table S1: Description of four meteorological stations.

| Station | Latitude | Longitude | Altitude (feet) |
| --- | --- | --- | --- |
| Multan | \| 30.1983 \| \| --- \| | \| \| 71.4697 \| \| --- \| \| \| --- \| --- \| | 397 |
| Bahawalpur | \| 29.4000 \| \| --- \| | 71.6833 | 370 |
| Barkhan | 29.8977 | 69.5256 | 3585 |
| Khanpur | \| 25.4626 \| \| --- \| \|  \| | \| 68.5912 \| \| --- \| | 45 |
